# Time-Dependent Evolution of Al–Al_4_C_3_ Composite Microstructure and Hardness during the Sintering Process

**DOI:** 10.3390/ma17194818

**Published:** 2024-09-30

**Authors:** Audel Santos Beltrán, Verónica Gallegos Orozco, Miriam Santos Beltrán, Hansel Medrano Prieto, Ivanovich Estrada Guel, Carmen Gallegos Orozco, Roberto Martínez Sánchez

**Affiliations:** 1 Departamento de Nanotecnología, Universidad Tecnológica de Chihuahua Sur, Km. 3.5 Carr. Chihuahua a Aldama, Chihuahua 31313, Mexico; msantos@utchsur.edu.mx (M.S.B.); hmedrano@utchsur.edu.mx (H.M.P.); 2 Departamento de Ciencias Básicas, Tecnológico Nacional de México, Campus Chihuahua, Ave. Tecnológico 2909, Chihuahua 31310, Mexico; 3 Centro de Investigación en Materiales Avanzados (CIMAV), Laboratorio Nacional de Nanotecnología, Miguel de Cervantes No. 120, Chihuahua 31136, Mexico; ivanovich.estrada@cimav.edu.mx (I.E.G.); roberto.martinez@cimav.edu.mx (R.M.S.); 4 Departamento de Económico Administrativo, Tecnológico Nacional de México, Campus Chihuahua II, Ave. de las Industrias #11101, Complejo Industrial Chihuahua, Chihuahua 31130, Mexico; carmen.go@chihuahua2.tecnm.mx

**Keywords:** metal–matrix composite, mechanical milling, nanoparticle dispersion

## Abstract

In this study, Al-Al_4_C_3_ compounds were manufactured by mechanical milling followed by heat treatment. To analyze the microstructural evolution, the composites were sintered at 550 °C at different sintering times of 2, 4 and 6 h. The mechanical results suggest that dislocation density and crystallite size primarily contribute to hardening before the sintering process, with a minimal contribution from particle dispersion in this condition. The compound exhibited a significant 75% increase in hardness after 2 h of sintering, primarily attributed to the nucleation and growth of Al_4_C_3_ nanorods. The HRTEM analysis, combined with geometric phase analysis (GPA) at and near the Al-Al_4_C_3_ interface of the nanorods, revealed strain field distributions primarily associated with partial screw dislocations and the presence of closely spaced dislocation dipoles. These findings are consistent with the microstructural parameters determined from X-ray diffraction pattern analysis using the convolutional multiple whole profile (CMWP) method. This analysis showed that the predominant dislocation character is primarily of the screw type, with the dislocation dipoles being closely correlated. Based on these results, it is suggested that samples with a lower weight percentage of reinforcement and longer sintering times may experience reduced brittleness in Al/Al_4_C_3_ composites. Strengthening contributions were calculated using the Langford–Cohen and Taylor equations.

## 1. Introduction

Metal matrix nanocomposites (MMNCs) have recently attracted considerable interest due to their potential applications in various fields, such as aerospace, transportation, electronics, and thermal management. These advanced materials are made by integrating nanoparticles, nanowires, nanotubes, or nanosheets into a metal matrix. The result is a remarkable strength-to-weight ratio and improved mechanical, physical, and chemical properties compared to traditional materials. The specific types of MMNCs vary depending on the metal matrix chosen, which typically includes metals such as aluminum, magnesium, titanium, copper, or iron. The development of MMNCs presents critical challenges, including achieving effective dispersion of nano-reinforcement phases, optimizing interface structure, and understanding the fundamental theoretical mechanisms governing mechanical and functional properties [1,2,3].

Mechanical milling (MM) is an effective method for synthesizing a wide variety of materials, including equilibrium, out-of-equilibrium, and nanocomposite materials. Mechanical milling offers a unique capability for fabricating materials that are challenging to produce using conventional methods. However, despite its versatility, several variables must be considered for optimal results, such as the type and size of the initial powders, the milling atmosphere, the ratio of the balls to the powder, the milling speed, the size of the balls, the milling time, and the process control agent [4]. To achieve a homogeneous distribution of nanometer-sized dispersoids in a ductile matrix, this method is critical. In particular, pure Al and its alloy matrix, reinforced with particles such as aluminum oxide (Al_2_O_3_), boron carbide (B_4_C), titanium carbide (TiC), silicon carbide (SiC), titanium dioxide (TiO_2_), titanium diboride (TiB_2_) and aluminum carbide (Al_4_C_3_), possess improved properties in tribology, mechanics, and corrosion [5]. Specifically, the Al_4_C_3_ intermetallic phase exhibits outstanding physical and mechanical properties, such as heat resistance, thermal cyclic resistance, wear protection, and low linear expansion. However, it has been reported that excessive Al_4_C_3_ could adversely affect the mechanical properties due to the intrinsic fragility of this phase [6]. Various techniques are commonly combined in the fabrication of Al/Al_4_C_3_ composite materials. These include mechanical milling (MM) followed by sintering, MM with extrusion, spark plasma sintering (SPS) combined with hot extrusion, SPS with MM, and hot pressing. Researchers have proposed multiple methods to integrate Al_4_C_3_ nanoparticles into the Al matrix, utilizing carbon-based starting powders such as graphite, fullerene, or graphene oxide (GO). The primary objective is to facilitate the precipitation of Al_4_C_3_ during heat treatment [7,8,9,10,11].

The mechanical properties of nanocomposite materials are influenced by the type and characteristics of the metal-reinforcing interface; therefore, it is crucial to identify the origin of heterogeneous microdeformations associated with these interfaces. It has been previously reported that the density of dislocations within metal–ceramic (M-C) interfaces becomes dominant throughout the crystal due to the high-volume fraction of these interfaces [12].

In order to adjust the parameters of the mechanical milling manufacturing process and obtain the required microstructure and mechanical properties, it is crucial to determine the relative contributions of reinforcement factors in composite materials [13]. The total strength of a material is derived from the cumulative effect of several factors, including grain limit reinforcement (σ_GB_), precipitation reinforcement (σ_p_), solid solution reinforcement (σ_ss_), Peierls–Nabarro friction (σ_PN_), and dislocation reinforcement (σ_d_). Each of these elements contributes to overall reinforcement, and their combined effect can be summarized as follows [14]:

σ_tot_ = σ_GB_ + σ_p_ + σ_p_ + σ_pn_ + σ_d_ (1)

The total hardness of a material can be determined by quantifying the various contributions using experimental results. Once calculated, this estimated total hardness can then be compared with the hardness measured experimentally for comparison and analysis.

The Convolutional Multiple Whole Profile (CMWP) fitting method has demonstrated its efficacy in determining the microstructural parameters such as dislocation densities (arrangements and character), and crystallite size distributions. CMWP fitting is performed using the CMWP software (http://csendes.elte.hu/mwp/. Accessed 26 June 2023) created by G. Ribárik and T. Ungár [15]. The examination of diffraction peaks using the CMWP software relies on microstructural models, assuming that the microstrains within the material matrix primarily result from the existence of dislocations. The observed diffraction pattern is fitted to a theoretically generated pattern, which takes into account the influence of dislocations (via average dislocation contrast factors) and the distribution of crystallite sizes. This fitting is achieved using five parameters: strain anisotropy parameter *q*, variance of the lognormal crystallite size distribution s_LN_, effective outer cut-off radius of dislocation ${R^{*}}_{e}$, dislocation density r, and crystallite size *L*_0_. These five parameters are represented as *a*, *b*, *c*, *d*, and *e*, respectively, in the CMWP software, as follows:

(2) q=a

(3) $$\sigma_{LN}=\frac{c}{\sqrt{2}}$$

(4) $$\rho =\frac{2}{\pi {\left(b_{Burgers}d\right)}^{2}}$$

(5) $${R^{*}}_{e}=\frac{\exp\left(-\frac{1}{4\;}\right)}{2e}$$

(6) $$L_{0}=\frac{2}{3}\exp\left(\frac{5}{4}c^{2}+b\right)$$

The *d* and *e* parameters are related to the dislocation density and effective outer cut-off radius of dislocations, respectively. The *q* value is the dislocation character (edge versus screw). The theoretical character of the dislocations *q* parameter is obtained from [16].

The theoretical *q* parameter is subsequently compared with the experimental *q* obtained from the CMWP program. If the value of the experimental *q* parameter closely aligns with the *q* value estimated for edge dislocations, then the dislocations are characterized as edge dislocations. Conversely, if the experimental *q* value closely matches the q value estimated for screw dislocations, then the dislocations exhibit a screw character, as described by T. Ungar et Al. Finally, if the experimental *q* value falls near the average of the edge and screw *q* values, the dislocations are considered to have a mixed character. The arrangement parameter *M* is expressed as [17,18]:

(7) $$M={R^{*}}_{e}\sqrt{\rho }$$

The dimensionless parameter $M=R_{e}\sqrt{\rho }\;$ describes the arrangement of dislocations, where the physical significance of *R_e_* (the effective outer cut-off radius of dislocations) aligns with that in the elastic stored energy of dislocations. *M* assumes a high value when dislocations are unrelated and randomly dispersed, causing their associated strain fields to extend over a significant distance. In such cases, *M* exceeds one (i.e., *M* > 1). Conversely, when dislocations of opposing signs are closely correlated and situated near each other, resulting in strain fields that extend over a short distance due to screening, *M* assumes a low value (*M* < 1) [15].

In our research, we synthesized Al_4_C_3_ powder through mechanical milling of aluminum and graphite, followed by thermal treatment. Our analysis focused on three key factors contributing to the strengthening of the aluminum matrix: dislocation density, crystallite size, and particle dispersion. Additionally, we compared the strain field distribution at the metal–nanorod interface with the microstructural parameters observed in CMWP.

## 2. Materials and Methods

The Al_4_C_3_ reinforcement powder was produced through a reaction between Al and C following the methodology outlined in our previous work [19]. The resulting powder (named mixture powder Mix) consists of approximately 51 wt.% Al_4_C_3_ particles with an average size of 13 nm, around 3 wt.% of C phase with an average size of 20 nm, and the remaining portion is composed of Al. The Al composites were produced by mixing Al powder (99.5% purity, Sigma-Aldrich, St. Louis, MO, USA) with 1 and 2 wt.% of Mix, each Al–Mix mixtures were mechanically milled in a high energy Simoloyer mill (Zoz GmbH, Wenden, Germany) for 8 h. Argon was used as the milling atmosphere and ~4 mL methanol as a process-control agent. The device and milling media used were made from hardened steel. The milling ball to powder weight ratio was set to 50:1. Consolidated samples of 6 mm diameter and 12 mm length were obtained by pressing the powder mixtures for 2 min at ~1250 MPa in uniaxial load. After that, the samples were sintered at different times: 2 h, 4 h and 6 h at 550 °C. Table 1 describes the nomenclature, composition and sintering times composites, the first number indicates the wt.% of the mixture powder M and the second one indicates the sintering time. The composites were studied by X-ray diffraction and Transmission Electron Microscopy (TEM). The diffraction profiles were measured by a Philips X’pert powder diffractometer using a Cu cathode (l = 0.15406 nm). The step size and step time were 0.02° and 5 s, respectively. The X-ray diffraction peak profile analysis was carried out to determine the crystallite size distribution and the dislocation density of the nanocomposites studied using the CMWP fitting procedure program. TEM characterization was performed using electron microscopy (JEOL Ltd. (Tokyo, Japan) JEM-2200FS equipped with a 200 kV field emission gun (FEG) and energy dispersive spectrometer (EDS) (JEOL Ltd., Seefeld, Germany)). For the preparation of TEM samples, a focused ion beam (JEM9320FIB) technique was used. Vickers hardness (HV) was measured by a Hardness tester (FM-07) (Future-Tech Corp., Wenden, Germany), using an indentation time of 10 s and a maximum load of 200 g.

## 3. Results

### 3.1. Microstructural Analysis

**Figure 1:**
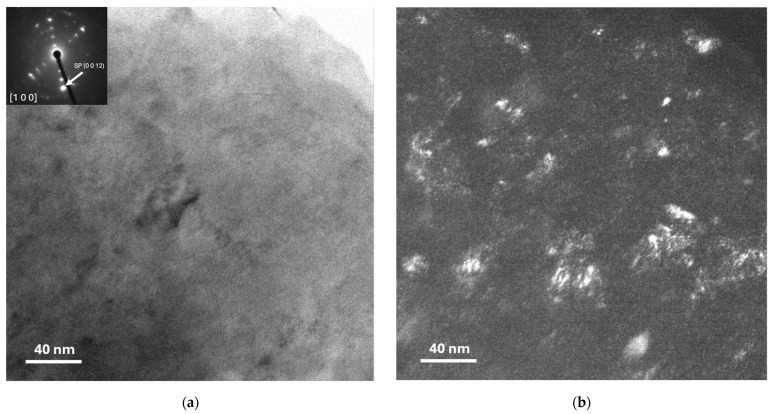
(**a**) TEM bright image with corresponding inset SAD pattern and (**b**) dark field image showing the distribution of the Al_4_C_3_ phase into the Al matrix.

Figure 1a,b display TEM bright-field and dark-field images, respectively. The corresponding selected area diffraction (SAD) patterns (visible in the inset) reveal the distribution of the Al_4_C_3_ phase within the Al matrix.

**Figure 2:**
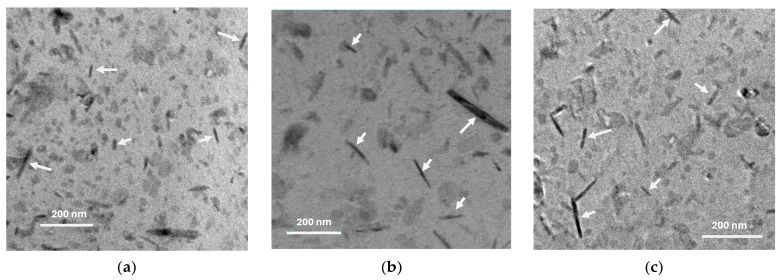
Bright-field TEM micrograph showing the Al_4_C_3_ nanorod dispersion on Al matrix (nanorods are indicated by white arrows) for (**a**) Al-22, (**b**) Al-24 and (**c**) Al26 samples.

The microstructural, morphological phase and dislocation strain distribution characteristics are revealed through the TEM-HRTEM imaging study. Figure 2a–c present the brightfield TEM images, illustrating the distribution of Al_4_C_3_ nanoparticles within the Al matrix, adopting a nanorod morphology (some of the nanorods are indicated by arrows for clarity).

**Figure 3:**
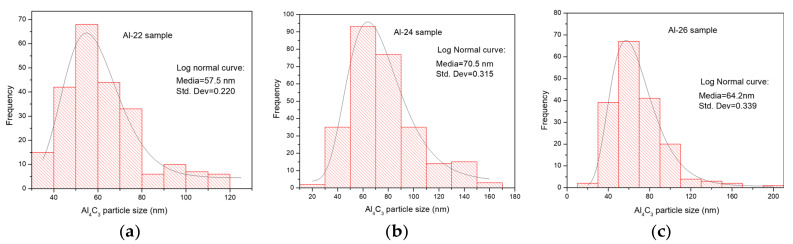
Nanorod size distribution histograms and fitting lognormal curve for (**a**) Al-22 sample, (**b**) Al-24 sample and (**c**) Al-26 sample.

Histograms with overlaid distribution curves of nanorod sizes, determined from TEM images for Al-22, Al-24, and Al-26 samples, are graphically represented in Figure 3a–c, respectively. The graphs also display the mean and standard deviation parameters obtained from histograms fitted to a log-normal function.

**Figure 4:**
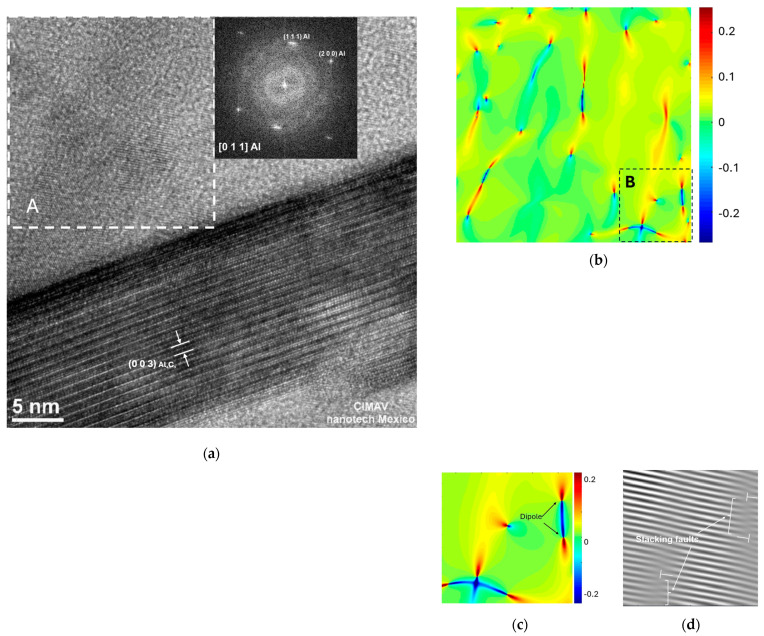
(**a**) High-Resolution Transmission Electron Microscopy (HRTEM) image of the Al-22 sample, highlighting selected area A, (**b**) distribution of strain fields within the specified region of selected area A, (**c**) amplified strain field distribution from selected area B and (**d**) Fourier-filtered image of selected area B, revealing only the fringes associated with the (111) lattice plane.

**Figure 5:**
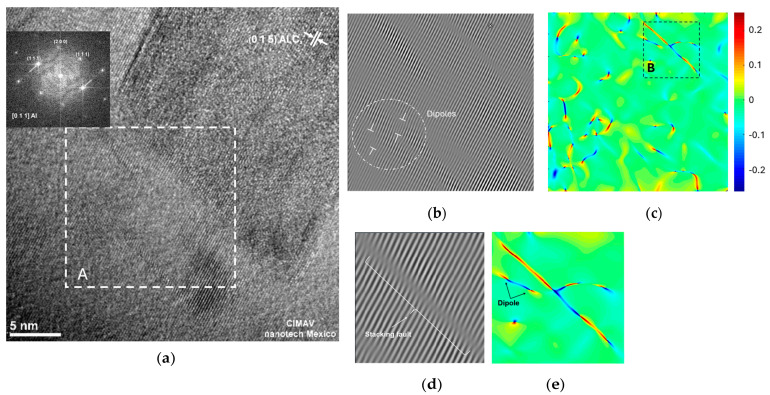
(**a**) HRTEM image of the Al-24 sample, highlighting selected area A and their corresponding digital diffraction pattern, (**b**) Fourier-filtered image of selected area A, revealing only the fringes associated with the (111) lattice plane, (**c**) distribution of strain fields within the specified region of selected area A, and (**d**,**e**) Enhanced view of Fourier-filtered and strain fields of specific area B.

**Figure 6:**
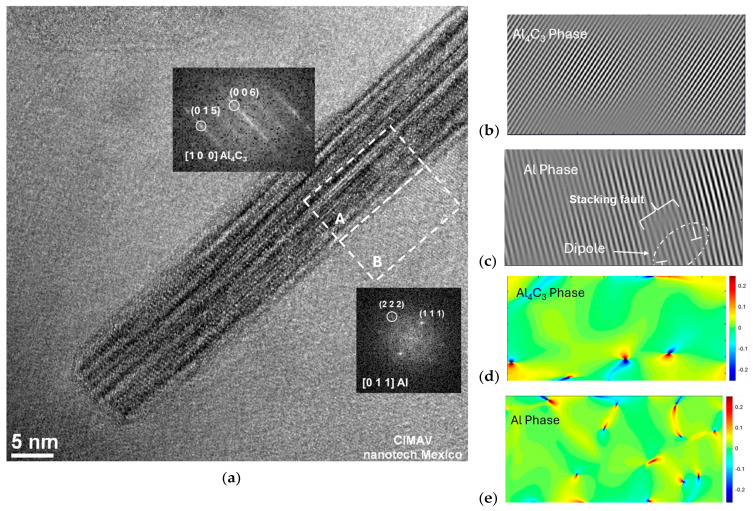
(**a**) HRTEM image of the Al-26 sample, highlighting selected areas A and B, and their corresponding diffraction patterns, (**b**) Fourier-filtered image of selected area A, revealing only the fringes associated with the (015) lattice plane of the Al_4_C_3_ phase, (**c**) Fourier-filtered image of selected area B, revealing only the fringes associated with the (101) lattice plane of the Al phase, (**d**) distribution of strain fields within the specified region of selected area A (Al_4_C_3_ phase) and (**e**) distribution of strain fields within the specified region of selected area B (Al phase).

High-resolution transmission electron microscopy (HRTEM) analyses were conducted in the vicinity of Al_4_C_3_ nanorods in samples with 2 wt.% of Mix sintered at intervals of 2, 4, and 6 h. Figure 4a, Figure 5a, and Figure 6a illustrate the selected region adjacent to the Al_4_C_3_ nanorod, along with the associated digital diffraction pattern based on the results of the Fast Fourier Transform (FFT) (obtained using GATAN software DigitalMicrograph 3.6.1, Gatan Inc.). The digital diffraction pattern conducted in Zone A on the Al-22 sample shows the Al phase oriented along the [011] direction. Using Geometric Phase Analysis (GPA), we determined the projected 2D strain tensor component, represented by ε, with the assistance of CrysTBox software (version 1.10) [20]

To calculate the geometric phase using the methodology proposed by M.J. Hytch and colleagues [21], we selected two primary reciprocal lattice vectors (g_1_(111) and g_2_(200)) corresponding to the Al phase from the digital diffraction pattern.

The strain field (ε_xy_) distribution within the specified region A is depicted in Figure 4b. This image reveals how the strain fields are influenced by the presence of dislocations within the examined region. In Figure 4c, we observe the enlarged FFT-filtered image obtained from the selected region B (see Figure 4b). The image shows the fringes associated with the (111) Al lattice plane. Figure 4d provides an enhanced view of the strain field (ε_xy_) distribution within the specified region B. The detailed relationship between lattice defects and strain fields (dislocations and stacking faults) is observed in Figure 4c,d.

The HRTEM image at the interface of an Al_4_C_3_ nanorod corresponding to the Al24 sample is shown in Figure 5a. The digital diffraction pattern inset obtained via FFT from region A indicates that this region corresponds to the aluminum Al phase, which is oriented along the [011] zone axis. The FFT-filtered image of region A using the (111) plane of the digital diffraction pattern, is shown in Figure 5b. This image illustrates the distribution of dislocations within the examined region. For calculating the geometric phase, two primary vectors in reciprocal space were chosen: g_1_ = 111 and g_2_ = 200 from de digital diffraction pattern. Strain field distribution (ε_xy_) within the specified region A is depicted in Figure 5c. Figure 5d provides an enhanced view of the specific area B of both Fourier-filtered and strain field images, detailing how the deformation fields are influenced by the presence of dislocations.

In Figure 6a, we observe the HRTEM image of an Al_4_C_3_ nanorod taken from the Al26 sample. The digital diffraction patterns corresponding to a region inside the nanorod (region A) and a region outside the nanorod (region B) are visible. Area A corresponds to the Al_4_C_3_ phase oriented along the [100] zone axis, while area B corresponds to the Al phase, oriented along the [101] zone axis. The Fourier-filtered images using the (015) plane for the Al_4_C_3_ phase (area A) and the (101) plane for the Al phase (area B), are shown in Figure 6b,c, respectively. The images display the dislocation distribution within the examined regions. For calculating the geometric phase, two primary vectors in the reciprocal space of the FFT image, were chosen: g_1_ = 015 and g_2_ = 006 and g_1_ = 021 and g_2_ = 101 for the Al_4_C_3_ and Al phase, respectively. The distribution of strain fields (ε_xy_) localized at the dislocations for Al_4_C_3_ and Al is depicted in Figure 6d,e, respectively.

Table 2 provides the following values obtained from X-ray diffraction patterns using the CMWP technique for each sample: the average dislocation density, *r*, the area average mean crystallite size, <*x*> area and the median and variance, *m* and s_LN_, of the log-normal size distribution function, the *q* experimental parameter, the average character number, *M* and the effective outer cut-off radius *R_e_* of dislocations.

The size distribution curve of crystallites was modeled using the log-normal distribution function, *f*(*x*), defined by Equation (8) [22]:

(8) $$f\left(x\right)=\frac{1}{\sqrt{2\pi \sigma }}\cdot \frac{1}{x}\cdot exp\left\{-\frac{{\left[\ln\left(x/m\right)\right]}^{2}}{2\cdot \sigma^{2}}\right\}$$

This function depends on the median, *m*, and the variance, *σ* parameters determined from X-ray analyses (see Table 2). The results corresponding to the concentration of 1 and 2% by weight of Mix and subjected to 0, 2, 4 and 6 h of sintering, are shown in Figure 3a,b, respectively.

### 3.2. Hardness Analysis

By expressing the microhardness (H) as the sum of each of the strengthening contributions in the Al composites, the following equation proposed by Cahn et al. [23] was used:

H = H_PN_ + H_SS_ + H_D_ + H_C_ + H_P_ (9)

where H_PN_ is the Peierls–Nabarro strengthening hardness contribution, H_SS_ is the contribution caused by solid solution, H_D_ is the dislocations contribution, H_C_ is the contribution by crystallite size, and H_P_ is the direct contribution by particle dispersion. Following the methodology outlined in prior studies [24], we computed the values of H_L_, H_D_, H_C_, and H_P_. Here, H_L_ represents the sum of H_PN_ and H_SS_. Table 3 presents the individual hardening contributions calculated using Vickers Hardness (VH) for each sample. Additionally, the table provides the experimental microhardness, H_EXP_ (VH), along with its corresponding standard deviation (SD).

## 4. Discussion

The discussion about classical mechanisms contributing to hardness, such as solid solution, Peierls–Nabarro, dislocation, grain size, particle dispersion and precipitation, can be found in our previous work [19,24]. During mechanical milling, numerous linear defects are induced in the aluminum matrix. These defects include dislocations generated by shear forces resulting from the impact of the milling media. In the early stages of milling, dislocations organize and form small-angle sub-boundaries. As the milling process advances, a nanostructured state composed of fine crystallites develops [25]. At this stage of the process, the aluminum matrix experiences an increase in its strength, mainly due to the reduction in crystallite size and the increase in dislocation density. On the other hand, during the sintering process, recovery, recrystallization, and grain growth are promoted, which negatively impact the mechanical properties of the composite.

**Figure 7:**
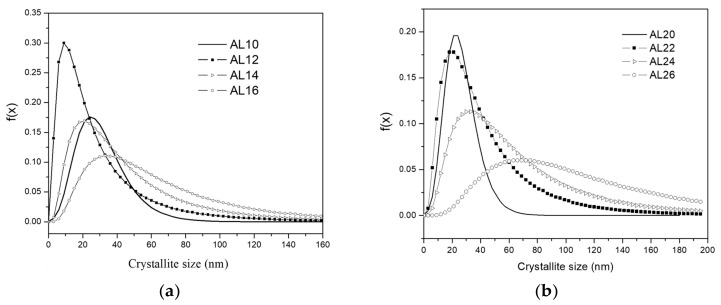
Log-normal grain size distribution from X-ray diffraction at 0, 2, 4 and 6 h of sintering time, for (**a**) Al-1 wt.% M and (**b**) Al-2 wt.% Mix composites.

**Figure 8:**
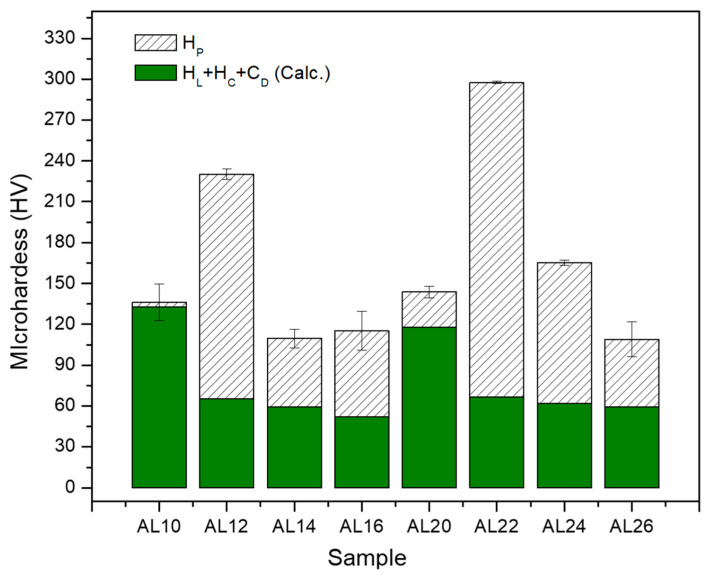
Calculated contribution microhardness H_L_, H_C_, H_D_, and H_P_ as a function of composition and sintering time in samples containing 1 and 2 wt.% of Mix.

Based on the results obtained from the CMWP, the logarithmic distribution of the normal grain size (as shown in Figure 7a,b) reveals a significant difference between the curves of the samples in the green state and those sintered for 2, 4, and 6 h (for both samples 1 and 2 wt.% of Mix). The findings indicate that the dispersion of crystallite size values increases with longer sintering times. On the other hand, the behavior of dislocation density concerning sintering time and the samples in the green state can be observed in Table 2. Specifically, in the green samples, relatively high dislocation density values were observed (~28 × 10^14^ m^−2^); however, in the sintered samples, a significant decrease is evident, with dislocation density values around ~1.8 × 10^14^ m^−2^ after 6 h of sintering in the samples containing 1 wt.% of Mix. Similar trends were observed in samples with 2 wt.% of Mix. In this study, the strengthening of the Al matrix was attributed to a combination of various mechanisms, including Peierls–Nabarro and solid solution strengthening (H_L_), dislocation density strengthening (H_D_), grain boundary strengthening (H_D_), and the Orowan effect (HP), as exposed in Equation (9). Based on the results obtained from the individual hardening contributions calculated (refer to Table 3), the value of the experimental microhardness is equated to the sum of three main contributions to hardening: H_L_ + H_D_ + H_C_ values. For more clarity, the graph in Figure 8 depicts the microhardness as a function of sintering time for samples containing 1 wt.% and 2 wt.% of Mix. The columns specify the calculated strengthening contribution, denoted as H_L_ + H_D_ + H_C_, as well as the contribution from particle dispersion, represented by HP. The latter is determined as the difference between the experimental microhardness (H_EXP_) and the sum of H_L_ + H_D_ + H_C_ (refer to Table 3). According to these findings, during mechanical milling, fine and irregular particles of Al_4_C_3_ disperse within the aluminum matrix, primarily contributing to the generation of dislocation density and reduction in crystallite size in the green state. It is noteworthy that for the samples in the green state (Al-10 and Al-20), an important contribution to hardening is attributed to dislocation density (H_D_) and crystallite size (H_C_), while a smaller contribution to hardening is observed due to Al_4_C_3_ nanoparticle dispersion, H_P_ (see Figure 8). These nanoparticles were observed using TEM conducted on the Mix powder, which was used to reinforce the Al matrix. Figure 1a,b present TEM bright-field and dark-field images, respectively, accompanied by the corresponding selected area diffraction (SAD) pattern (inset). These images reveal the distribution of the Al_4_C_3_ phase, which exhibits an irregular shape and ranges in size from approximately 5 nm to 20 nm.

On the other hand, after 2 h of sintering in both samples with 1 and 2 wt.% of Mix, a noticeable reduction in the combined effect of contributions (H_L_ + H_D_ + H_C_) was observed. Subsequently, a gradual decrease occurs at 4 and 6 h of sintering, as shown in the graph in Figure 8. However, the strengthening effect from particle dispersion (HP) intensifies after 2 h of sintering, reaching a dispersion strengthening value (HP) of approximately 160 HV (~80% higher than the green state sample, Al-10) in samples with 1 wt.% of Mix. In samples with 2 wt.% of Mix, a similar behavior was observed, with an increase of ~100% over the green state sample, Al-20. Moreover, a reduction was observed as the sintering time extended from 4 to 6 h, resulting in hardness values (HP) of approximately 60 HV for both samples with Mix contents of 1 and 2 wt.%. The results indicate that second phase precipitation with sintering significantly influences the Al matrix strengthening. Brightfield TEM images of the Al-22, Al-24, and Al-26 samples in Figure 2a–c, respectively, reveal a homogeneous dispersion of nanorod-like Al_4_C_3_ particles, which are responsible for the increase in hardness. On the other hand, the decrease in hardness of the aluminum matrix with sintering time is associated with the increase in nanorod size. Based on the histograms with overlaid distribution curves of nanorod sizes shown in Figure 3a–c, it becomes evident that as sintering time increases, there is an enlargement in the size of nanorods. The average media and standard deviation values obtained from histograms fitted to a log-normal function (values inset in each graph) increase from 57.5 (0.220) for the Al22 sample to 70.5 (0.315) for the Al24 sample, and finally to 64.2 (0.339) for the Al26 sample. In summary, during the mechanical milling (MM) process, fine and irregular Al_4_C_3_ particles are dispersed within the Al matrix. During the initial 2 h of sintering, these particles transform into Al_4_C_3_ nanorods, which continue to grow as the sintering period extends from 4 to 6 h. According to Lee et al., smaller particles grow at the expense of larger ones [26]. Consequently, this leads to an increase in the interparticle distance. The Orowan strengthening equation predicts a decrease in yield stress as the interparticle mean free path for dislocation motion increases [27]. The reduction in the strengthening dispersion effect (H_P_) particles with increasing sintering time, as depicted in Figure 8, can be reasonably attributed to the formation of larger particles, as expressed above.

For the design of this type of composite, it is essential to consider that short sintering times have a positive effect on the mechanical properties. However, it is crucial to ensure that the sintering time is sufficient for the carbon phase to fully transform into the Al_4_C_3_ phase. On the other hand, excessively long sintering times can cause excessive growth of the nanorods, which negatively impacts the mechanical properties. 

On the other hand, one of the issues encountered in metal matrix composites reinforced with ceramic particles is the generation of lattice dislocations near the interface, which can lead to the failure of a weak interface [28,29]. Various authors have reported the fragility of Al–Al_4_C_3_ composites [30]. The residual stresses are attributed to the mismatch of coefficient of thermal expansion (CTE) between the reinforcement and the Al matrix during the heating/cooling fabrication process and are directly related to the level of expansion coefficient mismatch between the matrix and reinforcement [31,32]. Previous investigations have reported various types of defects at the metal–ceramic interface, including edge and screw dislocations, planar defects such as twin boundaries and stacking faults, and low-angle twist boundaries, among others [33]. Other researchers have reported low-angle twist boundaries, which consist of a network of screw dislocations with the Burgers vector lying within the boundary plane [34,35,36].

From the analyses conducted using HRTEM and the GPA method near and at the nanorod interfaces, we observed strain field distributions primarily associated with partial screw dislocations accompanied by stacking faults. Additionally, edge dislocations with opposite signs, known as edge dislocation dipoles, were observed. The formation of edge dislocation dipoles is associated with the motion of screw dislocations containing dislocation jogs [37,38,39].

The HRTEM image analysis of the Al-22 sample revealed strain fields resulting from dislocations within the aluminum matrix near the nanorod (region A), as shown in Figure 4a,b. The image shows that the presence of these strain fields increased in areas near the nanorod interface. In the filtered image of Figure 4c, we observe typical dissociated screw dislocations composed of partial dislocations connected by an intrinsic stacking fault in the (111) plane. Figure 4d depicts the strain field corresponding to the filtered image, revealing the presence of dislocation dipoles.

Similar results were obtained from the HRTEM image analysis of the Al24 sample. In Figure 5b,c is observed the Fourier-filtered and strain fields of a specific area B, respectively. The filtered Fourier image (see Figure 5b) reveals the presence of dipoles in the Al phase near the nanorod, while Figure 5c illustrates the distribution of strain fields in this same region. Figure 5d and Figure 5e present magnified views of the filtered Fourier image and the strain fields in area B, respectively. The image details the presence of partial dislocations connected by a stacking fault. Figure 6 presents a comparative view of the Fourier-filtered and the strain field distribution images of the Al_4_C_3_ and Al phases at the Al–nanorod interface region of the Al-26 sample. In the filtered image of Figure 6b, there are virtually no defects observed in the atomic planes corresponding to the Al_4_C_3_ phase. In contrast, the Al phase region (Figure 6c) shows an intrinsic stacking fault in the (111) plane, resulting from dissociated screw dislocations. Figure 6d,e compare the strain fields in the Al_4_C_3_ and Al phases, respectively. A greater presence of deformation fields including dislocation dipoles is observed in the Al phase region. However, for the Al_4_C_3_ phase region, the strain fields are mainly located near the Al_4_C_3_-Al interface. Dislocation dipoles are better observed in the images of the strain field distribution. Dislocation dipoles were typically identified by a localized point of deformation, visible as a small blue and red area, connected by a thin red or orange band. In the strain field distribution, the green color in the strain images corresponds to the unstrained lattice plane, strains are positive and tensile in the red region, while the lattice is negative and compressive in the blue region.

These results are consistent with the parameter values derived from the CMWP adjustment. The experimental *q* parameter value, presented in Table 2, ranges between 1.03 and 1.58, while the calculated *q*, following the methodology described by T. Ungar et al., *q* = 1.31 is for pure screw dislocations and *q* = 0.33 for pure edge dislocations. These data lead to the interpretation that the predominant dislocation character within the samples is primarily of the screw type. Conversely, most samples exhibit an average M value of less than one, indicating a pronounced dipole character in the dislocations. This suggests a strong correlation with adjacent dislocations, leading to strain fields that are predominantly short-range due to the screening effect [15]. These results align with the analysis of the HRTEM images, in which closely correlated dipole dislocations are observed in close proximity to each other.

**Figure 9:**
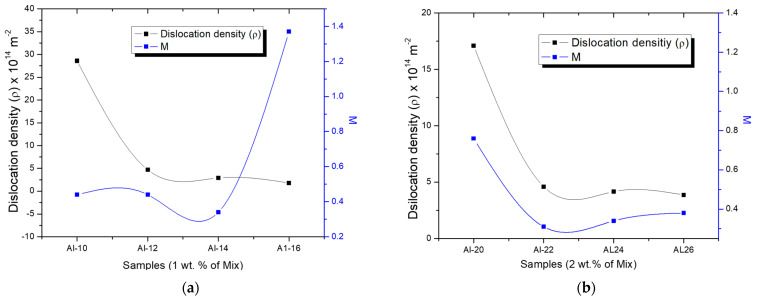
Results of dislocation density and the dipole character parameter at different sintering times for (**a**) 1 wt.% of Mix and (**b**) 2 wt.% of Mix.

The evolution of dislocation density as a function of sintering temperature is clearly observed in both graphs. The green state samples show high dislocation density values due to the mechanical milling process, reaching an average of 28 × 10^14^ m^−2^ and 17 × 10^14^ m^−2^ for the samples with 1 wt.% and 2 wt.% of Mix, respectively. However, a significant decrease is observed after 2 h of thermal treatment, attributed to recrystallization and restoration processes, with dislocation density dropping to approximately 5 × 10^14^ m^−2^ for both samples. These values remain practically unchanged after 4 and 6 h of sintering. This outcome is attributed to the competition between recrystallization and grain growth mechanisms, as well as the generation of dislocations caused by thermal mismatch between the matrix and the Al_4_C_3_ nanorods during sintering. On the other hand, as shown in both figures, for most of the samples in both the green and sintered states (1 and 2 wt.% of Mix), the dipole character parameter, M, is below 1, with values between 0.3 and 0.75 indicating a strong correlation between the dislocation dipoles in these samples. It has been reported by J.J. Gilman [37] that there is a direct relationship between dislocation dipole concentration and plastic strain, and that dipoles can also act as initiators of fracture. Also, the density of dislocation dipoles is often very high, which can significantly impact the mechanical properties of crystalline materials [40]. Therefore, the reported fragility of Al–Al_4_C_3_ composites may be related to the arrangement of dislocations (M). Samples that show an M < 1 indicate an accumulation of dislocation dipoles with a strongly screened strain field at the Al/Al_4_C_3_ nanorod interface, resulting in brittle behavior of the composite. On the other hand, the sample sintered for 6 h (Al-16 sample) showed a notable increase in the M value to 1.4 (see Figure 9a), suggesting that the dislocation dipoles are no longer closely correlated and instead display random dispersion and may exhibit reduced brittleness. This occurs because, under these conditions, the dislocation dipoles lack close correlation and are randomly dispersed at the Al/Al_4_C_3_ nanorod interface. This finding aligns with Gilman’s observation that the generation of dislocation dipoles causes hardening, while the annihilation of these dipoles leads to recovery.

Accordingly, achieving high hardening values in the aluminum matrix can be important for certain technological applications. Nevertheless, it is essential to emphasize the importance of developing Al/Al_4_C_3_ composites with reduced brittleness, without compromising their strength, for use in applications that demand such performance characteristics. Table 4 compares the mechanical properties (maximum tensile stress and microhardness) of the Al–Al_4_C_3_ composites obtained in this work with those reported by other authors in the literature. The results clearly indicate that the amount of Al_4_C_3_ nanoparticles dispersed in the Al matrix depends on the type of heat treatment, the amount of carbon, and the C phase employed. For example, when 7.5 wt.% of both C and fullerene were used, the carbon in the Al/fullerene composite was completely transformed into Al_4_C_3_, resulting in a microhardness of 292 HV. In contrast, the Al/graphite composite achieved a hardness of 188 HV, as the transformation to Al_4_C_3_ was only partial. In our work, the precipitation of Al_4_C_3_ nanoparticles was promoted because the initial powder mix (Mix) contained very fine, irregular Al_4_C_3_ particles. These particles were introduced into the aluminum matrix through mechanical milling (MM) and subsequently transformed into Al_4_C_3_ nanorods after relatively short sintering times.

## 5. Conclusions

Al–Al_4_C_3_ nanocomposites were synthesized through high-energy ball milling followed by a sintering process. The study found that a minimal concentration of Al_4_C_3_ and a brief sintering time were sufficient to effectively reinforce the aluminum matrix. The key findings of this study include:

- The strengthening effect, as determined from the CMWP and Taylor equations for the green state samples:

The strengthening contributions to the aluminum matrix from grain size and dislocation density were calculated based on microstructural analysis using X-ray diffraction (XRD) with the CMWP method. The contribution from particle precipitation was estimated as a residual effect. According to the results, in green state samples, dislocation density is the primary factor in the hardening of the Al matrix, followed by crystallite size, with particle dispersion contributing negligibly.

- The strengthening effect for samples sintered for 2, 4, and 6 h was evaluated using the CMWP and Taylor equations:

For samples sintered for 2 h, the primary strengthening mechanism is attributed to the nucleation and growth of Al_4_C_3_ nanorods (particle precipitation), along with increased dislocation density, leading to an approximate 75% increase in hardness. However, samples sintered for 4 and 6 h exhibited a significant decline in mechanical properties, primarily due to the excessive growth of the nanorods.

- HRTEM analysis combined with geometric phase analysis (GPA):

Analyses of the Al–nanorod interface revealed strain field distributions, primarily composed of edge dislocation dipoles formed by the movement of screw dislocations with jogs through the crystal lattice. The study found that screw dislocations were the dominant type within the samples, with closely correlated dipole dislocations located in close proximity to one another.

- Microstructural parameters determined from CMWP program:

Results from HRTEM and GPA analyses are consistent with the microstructural parameters, *q* (dislocation character, edge vs. screw) and *M* (dislocation arrangement), obtained from the analysis of X-ray diffraction patterns using the CMWP method. For most samples, the *q* parameter indicates that the dislocations are predominantly of screw character, while the *M* parameter showed values < 1, suggesting that the dislocation arrangement primarily consists of closely correlated dipoles. These findings imply that most Al/Al_4_C_3_ composites fabricated may exhibit high brittleness. However, samples with a lower weight percentage of the mix and longer sintering times, such as the Al-16 sample, exhibited an M parameter > 1, indicating that under these conditions, the dislocation dipoles are no longer closely correlated, which could result in reduced brittleness in Al/Al_4_C_3_ composites.

## Figures and Tables

**Table 1:** Compositions for studied Al–Al_4_C_3_ and samples nomenclature (in wt.%).

Nomenclature	Al (wt.%)	Mix, Mixture Powder (wt.%)	Sintering Time (h)
Al-10	99	1	0
Al-12	99	1	2
Al-14	99	1	4
A-16	99	1	6
Al-20	98	2	0
Al-22	98	2	2
Al-24	98	2	4
Al-26	98	2	6

**Table 2:** Microstructural parameters (*r*, *M*, *R_e_*, *<x>* area, *m* and s_LN_) obtained from the X-ray diffraction patterns using CMWP.

Composition	Dislocations *ρ* (10^14^ m^−2^)	<*x*>_area_ (nm)	*m* (nm)	*σ*	*q*	*M*	*R_e_* (nm)
Al-10	28.6	55.3	41.1	0.34	1.41	0.44	8.26
Al-12	4.7	172.5	21.0	0.91	1.56	0.44	20.33
Al-14	2.9	154.0	36.1	0.76	1.11	0.34	20.02
A1-16	1.8	184.3	55.0	0.69	1.58	1.37	102.38
Al-20	17.1	48.6	40.2	0.27	1.10	0.76	18.34
Al-22	4.6	139.2	34.2	0.75	1.55	0.31	14.50
Al-24	4.16	199.3	53.7	0.72	1.03	0.34	16.81
Al-26	3.87	271.8	101.5	0.62	1.29	0.38	19.72

**Table 3:** HL, HC, HD, HP and HEXP values in Vicker hardness (VH) for all samples.

Sample	H_L_	H_C_	H_D_	H_L_ + H_C_ + H_D_	H_P_ H_EXP_ − (H_L_ + H_C_ + H_D_)	H_EXP_	Std Dev.
A1-10	29.4	23.6	79.6	132.6	4.03	129.2	24.4
Al-12	25.3	7.5	32.4	65.2	−0.02	224.3	8.7
Al-14	25.3	8.4	25.5	59.2	2.6	115.8	12.3
Al-16	25.3	7.1	19.9	52.3	57.1	115.3	25.7
Al-20	29.4	26.8	61.6	117.8	44.3	143.8	7.8
Al-22	25.3	9.3	32.1	66.7	21.5	297.7	2.0
Al-24	25.3	6.5	30.4	62.2	43.3	165.3	3.4
Al-26	25.3	4.8	29.3	59.4	55.3	109.1	23.2

**Table 4:** Comparisons of mechanical properties of Al–Al_4_C_3_ composites with those from the literature.

Composition	Tensile Strength σ_max_ (Mpa)	Microhardness (VH)	Method	Refs.
Al-1/2 wt.% of Mix		~224/~298	MM and sintering	This work
Al-4 wt.% of Al_4_C_3_	~295	~40.2	MM and extrusion	[7]
Al-0.2/0.4 wt.% of Al_4_C_3_	166.2/183.1	~40/~45	SPS and Hot extrusion	[8]
Al~7.5 wt.% of C and 7.5 wt.% of Fullerene		~188/~292	SPS and MM	[9]
Al-5/10 wt.% of Al_4_C_3_	~190/300	~63 */~100 *	Hot pressing	[10]
Al-5/10 wt.% of C	400/600	~130 */~200 *	Hot pressing	[11]

* Calculated microhardness (VH) σ_max_/3 [41].

## Data Availability

The original contributions presented in the study are included in the article, further inquiries can be directed to the corresponding author.
